# Are there placebo or nocebo effects in balancing performance?

**DOI:** 10.1186/s41235-023-00476-z

**Published:** 2023-04-24

**Authors:** Áron Horváth, Attila Szabo, Vera Gál, Csilla Suhaj, Blanka Aranyosy, Ferenc Köteles

**Affiliations:** 1 Institute of Psychology, Károli Gáspár University of the Reformed Church in Hungary, Budapest, Hungary; 2 Institute of Psychology and Institute of Health Promotion and Sport Sciences, ELTE Eötvös Loránd University, 1117 Budapest, Hungary; 3 Doctoral School of Psychology, ELTE Eötvös Loránd University, Budapest, Hungary; 4 Institute of Psychology, ELTE Eötvös Loránd University, Budapest, Hungary

**Keywords:** Balance, Expectations, Placebo, Postural stability, Nocebo

## Abstract

Placebo and nocebo effects could influence the perceived, actual, or both postural stabilities. Therefore, this experiment examined whether postural stability is susceptible to placebo and nocebo effects. Driven by expectations, these cognitions could influence the motor stability of people in physical rehabilitation and those with motion instability. We randomly assigned 78 participants to a placebo, nocebo, or control group. Then, we applied a sham sports cream with positive, negative, or neutral instructions about its impact on balance. Next, we tested postural stability with a modified version of the Modified Clinical Test of Sensory Interaction in Balance, including standard, proprioceptive, visual, and vestibular tests before and after the intervention. Further, we measured expected and perceived performance with visual analog scales and assessed trait anxiety, change in state anxiety, optimism, holistic thinking, persistence, and cooperation with questionnaires. The intervention did not affect *actual test performances*; similarly, trait and state variables and expectations did not have an impact. Furthermore, the experimental manipulation and trait and state variables did not significantly affect perceived performance. However, the association between expectation and *perceived performance* was strong (*ϱ* = 0.627, *p* < 0.001). These findings suggest that postural stability is not susceptible to placebo and nocebo influences. Still, there is a dissociation between objective and subjective performance, showing that expectations impact perceived but not actual performance, which could fuel motivation in rehabilitation settings.

**Supplementary Information:**

The online version contains supplementary material available at 10.1186/s41235-023-00476-z.

## Significance statement

Postural stability is essential in locomotion, and it is critical in rehabilitation after accidents, sports injuries, or at an older age to avoid the risk of falls. Our research sought to establish whether placebo and nocebo effects, defined in this case as positive and negative suggestions, affect balance, the critical element of postural stability. We examined changes in four conditions (standard, proprioceptive, visual, and vestibular) before and after the respective intervention and presumed that the intervention would affect balancing performance. In this way, we adopted a *reverse strategy* in which results from applied research would generate research questions and hypotheses for basic research to determine the cognitive mechanisms of the expected effects. The results suggest that these balance tasks are not susceptible to placebo and nocebo effects. However, we used a mild external placebo/nocebo agent, an inert white cream. Still, a dissociation between actual (observed) and perceived (thought) performance was evident in our study. Accordingly, *expectations affected the perceived (subjective) but not the actual (objective) performance.* Based on (perceived) competence motivation, these findings could have implications for motivation in adherence to interventions aimed at improving postural stability. Indeed, if people believe that their performance is better due to an agent (in our case, an inert cream), they experience ‘*positive results*.’ These results are the rewards associated with an action (attending therapy). Therefore, they could reinforce the desired behavior and motivate the individual to adhere to physical therapy even though the objective (actual) results appear only later.

## Are there placebo or nocebo effects in balancing performance?

In many cases, believing or expecting something could be a self-fulfilling prophecy that leads to the predicted outcome. In a medical context, this process is called a placebo response if the expectation and the result are positive (i.e., beneficial) (Benedetti, 2009) and a nocebo response if the anticipation and the outcome are negative (i.e., harmful or unpleasant; (Hahn, 1997, 1999). For example, when somebody receives a pharmacologically inert treatment with information about the expected improvements and side effects, they may experience and report the predicted consequences. There are at least three main mechanisms that can drive placebo and nocebo responses: conscious expectations (Kirsch, 1997), classical or social conditioning (Benedetti, 2009), and the so-called meaning response (Moerman, 2002a, 2002b). Placebo and nocebo responses may manifest as peripheral physiological or behavioral changes (de la Fuente-Fernández et al., 2001). Still, in most cases, they dominantly influence the subjective experience (i.e., the perception of the physical condition or performance) without any peripheral or behavioral change (Köteles, 2021; Spiro, 2000).

Motor performance is highly susceptible to top-down influences (Bérdi et al., 2011; Horváth et al., 2021; Hurst et al., 2019). On a neural level, it was revealed that placebo and nocebo responses could modify the activity of the opioid, endocannabinoid, and dopamine neurotransmitter systems that regulate pain, fatigue, motivation, and arousal, i.e., factors that can substantially impact motor performance (Beedie et al., 2020). Several empirical studies support this idea; for example, Benedetti et al. (2007) administered morphine in the preparation period for endurance runners, decreasing pain and increasing physical performance due to its analgesic effect. However, on the day of the competition, only a placebo was administered, which improved athletes’ performance. Placebo and nocebo effects were also demonstrated in sprint performance (Beedie et al., 2007) and maximal voluntary strength exercise (Emadi Andani et al., 2015; Tallis et al., 2016). To enhance the performance of their athletes, a considerable proportion of sports trainers use placebos; Szabo and Müller (2016) found that 44% of coaches have administered a placebo to their athletes, while Brooling et al. (2008) reported a rate as high as 62%.

Despite the high number of studies showing the importance of placebo and nocebo effects in motor performance, there are contradictory and ambiguous findings too. For example, a sham sports drink administered with nocebo instruction did not decrease actual performance (i.e., peak minute power during incremental arm crank ergometry) but increased perceived exertion (Bottoms et al., 2014). In contrast, the placebo drink increased actual performance and, at the same time, made perceived exertion lower. Hurst et al. (2017) gave participants an inert capsule with placebo or nocebo instruction. Their results showed that nocebo treatment negatively influenced sprint performance; moreover, placebo was beneficial only for those *intending* to use sports supplements. In contrast, Corsi et al. (2019) found that nocebo instruction negatively affected maximal strength, while placebo instruction did not have a positive effect.

These equivocal findings suggest that the development of placebo and nocebo effects can at least partly depend on different factors. For example, the type of the agent (e.g., ergogenic aid, sham transcranial magnetic stimulation) and the entire intervention play an important role (Hurst et al., 2019). The study's design, the characteristics of the investigated population, and the exact aspect of motor performance may also influence the outcome (Horváth et al., 2021). Psychological factors can also play a role in developing the placebo and nocebo response, often in interaction with the intervention. Kern et al. (2020) found that optimism was associated with a more robust placebo response, while pessimism, fear, and anxiety were associated with an enhanced nocebo response (person-treatment interactions). Also, holistic thinking style and spirituality appear to increase the proneness to placebo and nocebo reaction, typically in interaction with the type of placebo intervention (Hyland, 2011). We know only one study investigating the role of personality traits in response to placebo and nocebo interventions concerning motor performance. Corsi et al. (2016) found that the magnitude of nocebo response (force production) was associated with lower levels of optimism and higher levels of anxiety. Also, people with higher levels of persistence perceived the negative effect as lower. In this study, the behavioral nocebo response was impacted by the effectiveness of the experimental manipulation (i.e., the information given with the inert treatment), which shows that conscious expectations can play a role in sports performance that partly relies on voluntary effort.

In summary, empirical evidence suggests that (1) the contribution of placebo/nocebo effects to motor performance might be substantial; however, (2) it is often determined by the interaction of multiple factors, including personality characteristics. An individual’s response to a placebo/nocebo treatment is difficult to predict, which limits the usability of placebo interventions (Feltner et al., 2009; Kaptchuk et al., 2008). Understanding the underlying factors and their interactions can improve the predictability of the response, which in turn might impact the applicability of placebo interventions not just for athletes but also for patients with Parkinson’s disease (Benedetti et al., 2003). In the same vein, it can help us to identify individuals with an above average malleability to nocebo effects in order to prevent those negative effects; for example, the nocebo effect can negatively impact muscle strength in surgical patients (Zech et al., 2020).

Balancing ability, such as maintaining an upright stance despite minor kinematic disturbances or control errors (O’Connor et al., 2016), plays an essential role in many areas of life. For older adults, worse balancing ability is associated with a higher risk of falls (Lord & Sturnieks, 2005; Maki et al., 1994), which may result in serious injuries. In athletes, a better balancing ability is often associated with better sports performance (Hrysomallis, 2011). Despite its practical importance, only two empirical studies have addressed the malleability of the balancing ability to placebo/nocebo interventions to date. In the study of Villa-Sánchez and et al. (2019), an inert intervention carried out using an electrical device administered with a placebo instruction led to an improvement in actual balancing ability in a single-leg stance task. Beyond actual performance, participants in the placebo group reported higher performance-related expectations and better perceived postural stability than the control group. As the study investigated the placebo effect, no nocebo condition was included. Also, single-leg stance is an unusual posture; thus, it is not clear how performance in such a task can be generalized to everyday situations. In a more recent work, using an ecologically more valid design, including a placebo, a nocebo, and a control groups, assessment of stability of bipedal stance, and an inert pill administered with placebo or nocebo instructions, the predicted changes in expectations, actual (center of pressure metrics), and perceived performance in bipedal and unipedal conditions were found (Russell et al., 2022). The latter study was designed and conducted simultaneously with the study reported in this paper. Authors of both previous studies concluded that (1) balancing ability is sensitive to placebo/nocebo interventions, and (2) the effect is mediated by expectation. It is worth noting that the sample size was comparatively small for both studies, which can statistically bias the results (Button et al., 2013).

### Aims and hypotheses

In this laboratory investigation, we aimed (1) to better understand the role of expectation on balancing performance (including possible interactions between expectation and various sensory modalities that contribute to balancing) and (2) to extend previous findings by including trait/state psychological factors that might interact with the placebo/nocebo interventions. We adopted an exploratory applied approach through which any hypothesized effects would be subject to further scrutiny via more basic research.

We expected that an inert treatment (a claimed "sports cream" without an active agent applied on the skin of the legs), administered with positive instruction (placebo), would evoke a positive expectation and will have a positive effect on actual and perceived postural stability. In contrast, the same inert treatment with adverse instruction (nocebo) will evoke a negative expectation and negatively affect real and perceived postural stability. We also expected person-treatment interactions, such as the possible role of optimism, holistic thinking, state and trait anxiety, physiological stress, and persistence in placebo or nocebo effects.

## Methods

This research protocol was preregistered at the Open Science Foundation (www.osf.io; https://doi.org/10.17605/OSF.IO/YUQ7K). The data, syntax, and results of the analyses are accessible at: https://osf.io/6fbxs/.

### Participants

Participants were first year psychology students recruited through a university course at Eötvös Loránd University, Hungary. They got partial course credit for their participation and had to be at least 18 years old without injury or pain in the lower limbs. A priori sample size calculation indicated that at least 78 participants were required to reach an alpha level of 0.05 and a power of 0.8, with an effect size of *f* = 0.180 to test a two-way interaction in a mixed analysis of variance (ANOVA; one between-subject factor with three levels and one within-subject factor with two levels). The effect size was based on the systematic review of Hurst et al. (2019); we used the G*Power software v3.1.9.4 developed by Erdfelder et al. (1996). Six participants were excluded because they reported severe pain or injury in their lower limbs. The experiment was approved by the Research Ethics Committee of the Faculty of Education and Psychology, Eötvös Loránd University (permission number 2021/372). All participants signed an informed consent form before the experiment; they also confirmed that they were not under the influence of alcohol or any psychoactive substances and were not being or were treated for a neurological or psychiatric condition. The final sample consisted of 78 participants (57 female, mean age: 20.7 ± 3.3 yrs.). Participants were randomized into three experimental groups (placebo, nocebo, or control group), with simple randomization in a 1:1:1 ratio, with the “sample” function of the R programming language (R Core Team, 2020).

### Postural stability

Postural stability was measured with the BTrackS™ Balance Plate (O’Connor et al., 2016) using the modified Clinical Test of Sensory Integration and Balance protocol (Goble et al., 2019). In order to assess to contribution of various sensory modalities involved in balancing ability, four different variations of the test were conducted: eyes open stable surface (“standard”; integration of all available modalities), eyes open unstable surface (“vision”; primary reliance on visual information, limited proprioceptive information), eyes closed stable surface (“proprioception”; primary reliance on proprioceptive information), and eyes closed unstable surface (“vestibular”; reliance on vestibular information due to lack of visual information and limited proprioceptive information). Participants were asked to stand on the device barefoot in a tight straddle stand (the required position of the two feet were indicated on the device) with hands on the hips. They received the information that the task was standing as stable as possible during the trials. Each task variation involved one practice trial and three tests trials, with each trial lasting 20 s. We used the Total COP Path Length index, provided by the BTrackS software (Goble et al., 2019), as the indicator of postural stability (Hearn et al., 2018) and calculated the mean of the three test trials within one test. Higher values refer to worse postural stability. Internal reliability of the scores was good for every variation. (Cronbach’s alphas ranged from 0.79 to 0.95.)

### Questionnaires

#### Expected change in performance

To assess the expected change in postural stability after the experimental manipulation, we used a 100-mm Visual Analogue Scale (VAS), where participants had to rate “*In what direction and to what extent will your performance change?*”. The scale started with “*will be the worst possible*” and ended with “*will be the best possible*” captions, the middle point was captioned with “*will not change*”.

#### Perceived change in performance

Perceived change in postural stability after the experimental manipulation was also assessed with a 100-mm VAS, where participants had to answer the question “*In what direction and to what extent did your performance change?*”. The scale started with “*became the worst possible*”, and ended with “*became the best possible*”, the middle point was captioned with “*did not change*”.

#### State and trait anxiety

Participants completed the Hungarian version of the State-Trait Anxiety Inventory (STAI) (Sipos & Sipos, 1983). The trait version measures the general extent of anxiety. It consists of 20 questions (e.g., “*Some unimportant thought runs through my mind and bother me*”), rated on a 4-point Likert scale; higher values refer to higher levels of trait anxiety. Internal consistency of the scale was excellent in our sample (Cronbach’s alpha = 0.92). The state version of the questionnaire assesses the acute level of anxiety with 20 questions (e.g., “*I am tense*”). State anxiety was measured both before and after the intervention. Internal reliability was excellent in both cases (Cronbach’s alpha = 0.93 for both scales). To calculate the change in state anxiety, we subtracted the pre-intervention scores from the post-intervention scores. Higher values referred to increased anxiety levels after the intervention.

#### Optimism

Dispositional optimism was measured with the Hungarian version of the LOT-R questionnaire (Bérdi & Köteles, 2010). The questionnaire consists of 10 overall questions (e.g., “*I’m always optimistic about my future*”), including four filler items, that had to be answered on a five-point Likert scale. Higher total scores refer to a higher level of dispositional optimism. Internal consistency in our sample was good (Cronbach’s alpha = 0.88).

#### Holistic thinking

Holistic thinking about health was measured with the Holistic Health subscale of the Hungarian version of the Holistic Complementary and Alternative Medicine Questionnaire (HCAMQ-H) (Köteles, 2014). Participants had to rate statements (e.g., “*Positive thinking can help you fight off a minor illness*”) on a six-point Likert scale, where higher values refer to a higher level of holistic thinking about health. In our sample, the internal reliability of the scale was acceptable (Cronbach’s alpha = 0.73).

#### Persistence

Persistence was measured with the Persistence subscale of the TCI55 (Hungarian Short Form of the Cloninger Temperament and Character Inventory (Szabó et al., 2016). It consisted of two items that had to be answered on a 4-point Likert scale, where higher values referred to higher persistence. The internal reliability of the questionnaire was good in our sample (Cronbach’s alpha = 0.79).

#### Motivation to cooperate

Participants’ motivation to cooperate was measured with a 6-item scale (Szemerszky et al., 2010). Participants rated statements (e.g., “*I am happy to participate in this study*”) on a 5-point Likert scale, where higher values referred to a higher motivation to cooperate. This scale was used to control systematic bias based on participants’ (conscious and non-conscious) intention to help the experimenters. In this study, the internal reliability of the instrument was good (Cronbach’s alpha = 0.83).

### Procedure and experimental manipulation

After arriving at the laboratory, participants filled out a short questionnaire consisting of demographic questions and STAI state version (pre-intervention). In the next step, they were randomly assigned to one of the experimental groups (control/placebo/nocebo) and completed the pre-intervention postural stability measurements in a randomized order. Subsequently, participants were asked to listen to the standardized group-specific verbal instruction about the effect of the claimed sports cream through headphones; this method was chosen to make sure that everyone within a group received identical information. The placebo group received the information that the cream *improves* balancing ability as it facilitates warming-up of the muscles. In contrast, the nocebo group was told that the cream *decreases* balancing ability primarily due to a slight anesthetic effect. Finally, the control group was informed that the cream *does not affect* balancing ability (for the exact instructions, see Additional file 1). We chose this agent to avoid any potential harm associated with ingestible substances, e.g., systemic nocebo effects, such as headache or nausea.

**Fig. 1:**
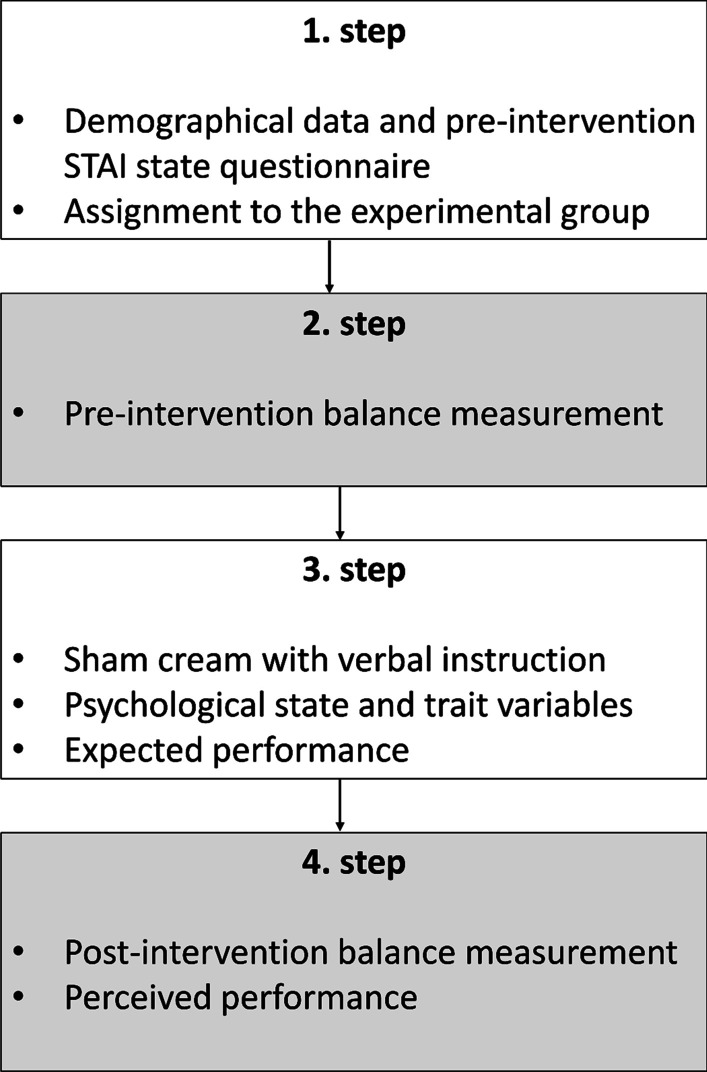
Flowchart of the experiment. Note: 2. and 4. step was carried out in a separate room by a blinded experimenter

Participants were asked to spread the inert cream on both of their legs from the ankle to the knee area with 3 g per leg; the experimenter checked if participants applied the cream as instructed. The cream was *unguentum stearini*, a neutral cream, available throughout pharmacies in Hungary without a prescription and does not contain any active ingredients. Subsequently, they completed the STAI state (post-intervention) and STAI trait, LOT-R, Holistic Thinking subscale of the HCAMQ, Motivation to Cooperate, and TCI55 questionnaires and rated the expected change in performance. Finally, they completed the post-intervention postural stability measurements in a randomized order and indicated their perceived change in performance. The postural stability measurements (both pre- and post-intervention) were performed by a blinded experimenter who was not aware of the participants’ group assignment in a separate room (Fig. 1).

## Results

### Descriptive statistics

We used the SPSS v27 software to conduct the data analysis (IBM Corporation, New York). Descriptive statistics of the investigated variables on a group level are presented in Table 1.

**Table 1:** Descriptive statistics of the investigated variables for all participants

	Mean	Std. Deviation	Minimum	Maximum
Expected change in performance	5.26	1.598	1.7	9.8
Perceived change in performance	5.00	1.529	1.8	9.0
STAI trait	45.01	10.721	23.00	69.00
STAI state pre- and post-difference	0.94	3.805	−8.00	12.00
Optimism	22.19	5.809	7.00	30.00
Motivation to cooperate	25.88	3.629	6.00	30.00
Holistic thinking	27.45	2.913	14.00	30.00
Persistence	5.29	1.604	2.00	8.00
Postural stability Standard pre	15.90	5.653	6.17	38.15
Postural stability Standard post	15.10	5.518	6.85	44.36
Postural stability Proprioception pre	20.18	6.187	6.87	38.00
Postural stability Proprioception post	20.21	5.898	6.47	34.27
Postural stability Vision pre	23.51	7.247	13.78	59.53
Postural stability Vision post	22.72	6.636	11.89	44.48
Postural stability Vestibular pre	50.03	13.049	14.74	80.72
Postural stability Vestibular post	47.21	14.563	11.89	81.86

Descriptive statistics of the performance indices split by experimental groups are presented in Table 2.

**Table 2:** Performance indices of the investigated variables by experimental groups

Test	Measurement	Group	Mean	Std. deviation
Expected change in performance		Placebo	5.97	1.247
		Nocebo	4.59	1.945
		Control	5.21	1.243
		Total	5.26	1.598
Perceived change in performance		Placebo	5.84	1.516
		Nocebo	4.61	1.428
		Control	4.54	1.326
		Total	5.00	1.529
Standard	Pre	Placebo	16.15	6.089
		Nocebo	16.82	6.483
		Control	14.72	4.115
		Total	15.90	5.689
	Post	Placebo	15.34	6.777
		Nocebo	15.98	5.350
		Control	13.97	4.200
		Total	15.10	5.530
Proprioception	Pre	Placebo	20.85	6.003
		Nocebo	20.33	7.273
		Control	19.37	5.271
		Total	20.18	6.187
	Post	Placebo	20.79	6.193
		Nocebo	20.67	6.359
		Control	19.18	5.164
		Total	20.21	5.898
Vision	Pre	Placebo	23.42	9.873
		Nocebo	25.27	5.766
		Control	21.81	4.974
		Total	23.51	7.247
	Post	Placebo	22.47	7.603
		Nocebo	24.73	5.489
		Control	20.97	6.342
		Total	22.72	6.636
Vestibular	Pre	Placebo	50.43	13.81
		Nocebo	51.05	11.077
		Control	48.60	14.413
		Total	50.03	13.0489
	Post	Placebo	47.59	14.484
		Nocebo	48.03	11.522
		Control	46.01	17.560
		Total	47.21	14.563

### Manipulation check

The effectiveness of experimental manipulation was checked using a one-way between-subject ANOVA (condition: placebo, nocebo, or control) with expected performance as the outcome variable. In most of the cases, the outcome variables were not normally distributed. In the study preregistration, we claimed that we would use transformation in this case. However, we decided not to do that, as different variables may require different transformations, making the investigation of the within-subject effects difficult. We used Bonferroni correction for post hoc comparisons with *p* < 0.05 as the accepted level of significance throughout the analysis (Note: the preregistered Tukey test could not be conducted, as SPSS v27 software does not provide an option for that). To improve comparability with earlier findings, partial eta squared values were converted to Cohen’s d values using an online effect size calculator (https://www.psychometrica.de/effect_size.html).

We found a significant main effect of condition (*F*(2,75) = 5.355), *p* = 0.007, *d* = 0.756). *Post hoc* test showed a significant (*p* = 0.005) difference only between the placebo and the nocebo groups (i.e., the control group did not differ significantly from the placebo and the nocebo group, *p* = 0.239 and *p* = 0.419, respectively).

### Postural stability (actual performance)

To test the effect of the intervention on postural stability performance, we conducted four 2 (within-subject; time: pre- or post-intervention) × 3 (between-subject; condition: placebo or nocebo or control group) mixed analyses of covariance (ANCOVA) for the four postural stability tests (standard, visual, proprioceptive, vestibular); the outcome variable was actual postural stability. The covariates in the model were: change in state anxiety, trait anxiety, holistic thinking, motivation to cooperate, persistence, optimism, and expected change in performance. (Note: Assessment of heart rate and rate variability was also preregistered for the study but not included in the analysis due to technical issues, i.e., length of the assessed sessions and motor activity during the balancing tasks did not allow for reliable calculation of heart rate and heart rate variability).

**Fig. 2:**
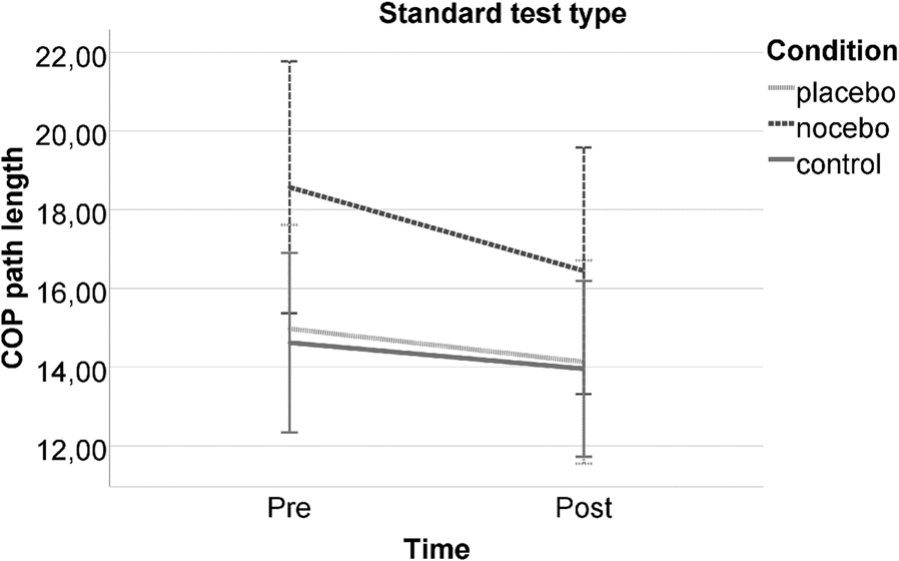
Postural stability performance of the experimental groups pre- and post-intervention in the standard test type. Note: The error bars reflect 95% CI

For the standard postural stability test, no time*condition interaction (*F*(2,54) = 0.698, *p* = 0.502, *d* = 0.320), or condition (*F*(2,54) = 1.693, *p* = 0.194, *d* = 0.501), but a significant (*F*(1,54) = 5.563, *p* = 0.022, *d* = 0.640) time main effect was observed (Fig. 2), the post-intervention test showing a better performance (lower COP path length). No significant interactions with covariates were found.

**Fig. 3:**
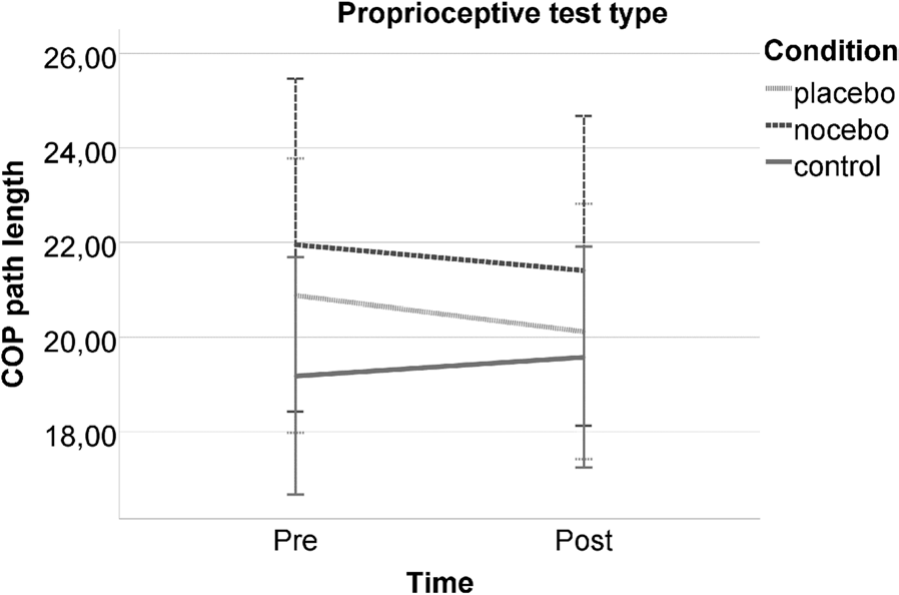
Postural stability performance of the experimental groups pre- and post-intervention in the proprioceptive test type. Note: The error bars reflect 95% CI

For the proprioceptive test type, no time*condition interaction (*F*(2,54) = 0.328, *p* = 0.722, *d* = 0.220), no time main effect (*F*(1,54) = 0.191, *p* = 0.663, *d* = 0.127), and no condition main effect (*F*(2,54) = 0.759, *p* = 0.473, *d* = 0.330) was observed (Fig. 3). However, the condition*time*persistence interaction was significant (*F*(3,54) = 4.143, *p* = 0.010, *d* = 0.959). Persistence showed a positive, medium level association with COP path length only in the pre-intervention measurement for the control group (*rho* (*ϱ*) = 0.425, *p* = 0.030); correlation was not significant in all other cases.

**Fig. 4:**
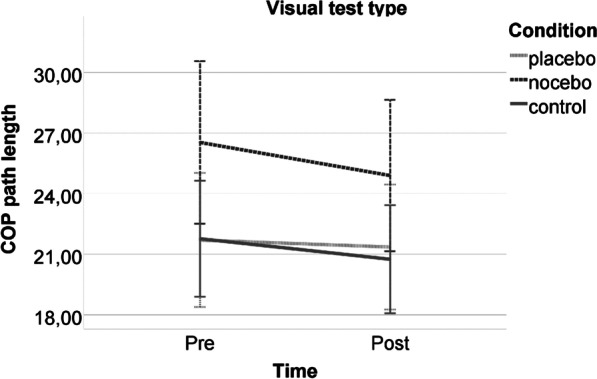
Postural stability performance of the experimental groups pre- and post-intervention in the vision test type. Note: The error bars reflect 95% CI

For the visual test type, no time*condition interaction (*F*(2,54) = 0.199, *p* = 0.821, *d* = 0.168), no time main effect (*F*(1,54) = 1.602, *p* = 0.211, *d* = 0.346), and no condition main effect were found (*F*(2,54) = 2.348, *p* = 0.105,* d* = 0.590) (Fig. 4). No significant interactions with covariates were found.

**Fig. 5:**
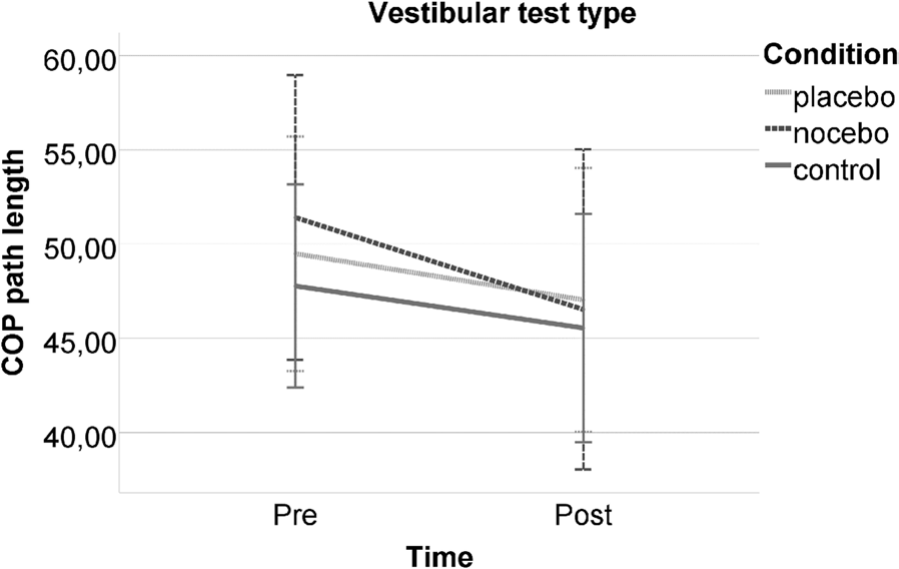
Postural stability performance of the experimental groups pre- and post-intervention in the vestibular test type. Note: The error bars reflect 95% CI

For the vestibular test type, no significant time*condition interaction (*F*(2,54) = 0.283, *p* = 0.755, *d* = 0.201) and no significant condition main effect (*F*(2,54) = 0.153, *p* = 0.859, *d* = 0.155) were found (Fig. 5). However, a significant time main effect was observed (*F*(1,54) = 4.638, *p* = 0.036, *d* = 0.586), where the post-tests showed a better performance. Concerning the covariates, only the time*condition*optimism interaction was significant (*F*(3,54) = 3.031, *p* = 0.037, *d* = 0.820). Optimism showed a significant, negative, medium level correlation with COP path length index in the pre-intervention measurement in the placebo group (*rho *(*ϱ*) = −0.437, *p* = 0.026), and in the post-intervention measurement in the nocebo group (*rho *(*ϱ*) = −0.502, *p* = 0.009), and no significant association for the other cases.

### Perceived performance

**Fig. 6:**
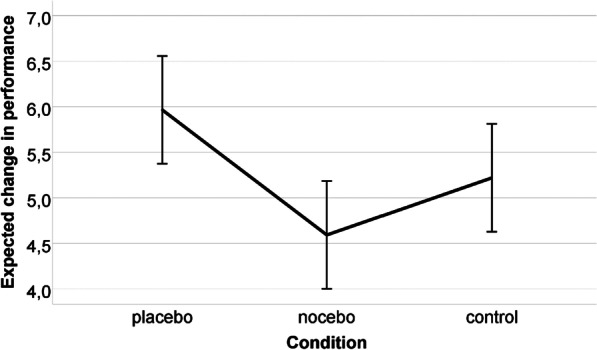
Perceived performance of the three experimental groups. Note: The error bars reflect 95% CI

**Fig. 7:**
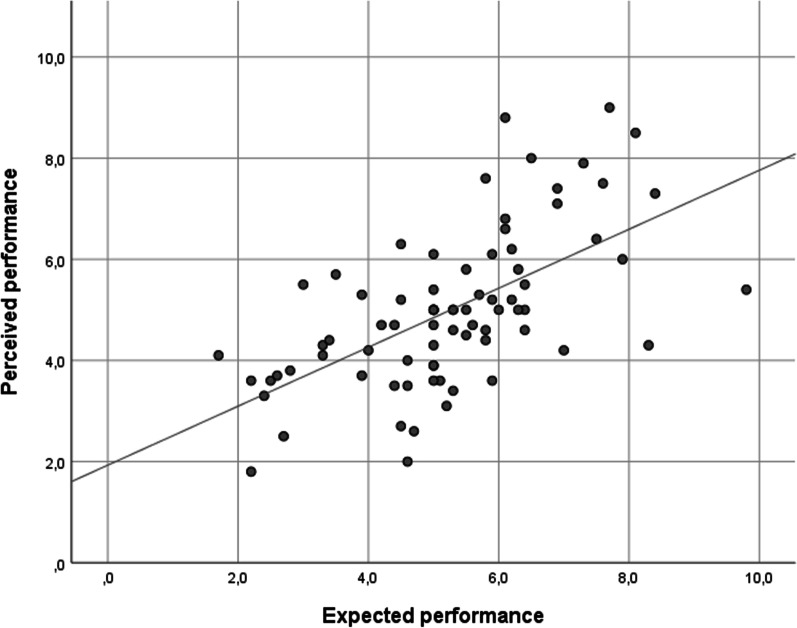
The association between expected and perceived performance

To investigate the effect of the intervention on perceived performance, we conducted an ANCOVA with one between-subject factor (condition: placebo, nocebo, or control group) with the same covariates as for actual performance. No significant condition main effect was revealed (*F*(2,54) = 1.730, *p* = 0.187, *d* = 0.505) (Fig. 6). The effect of expected performance was significant (*F*(1,54) = 21.156, *p* < 0.001, *d* = 1.250). Irrespective of the experimental condition, expected and perceived performance showed a significant, strong, positive association (*rho *(*ϱ*) = 0.627, *p* < 0.001) (Fig. 7). No other covariates or conditions by covariate terms showed a significant effect.

### Post hoc analyses

ANOVAs without the covariates.

As an explorative analysis, we conducted all analyses without the covariates (the results can be found in the SPSS output file at https://osf.io/6fbxs/). For actual performance, no significant time*condition interaction was observed for any of the tests; thus, these results also showed that the experimental manipulation did not affect postural stability. For perceived performance we found a significant condition main effect (*F*(2,78) = 6.828, *p* = 0.002, *d* = 0.853). According to the post hoc tests, the placebo group showed significantly higher mean than the nocebo (*p* = 0.008) and the control (*p* = 0.005) group, but there was no difference between the control group and the nocebo group (*p* = 1.000).

Association between perceived and actual change in performance.

To test the association between actual and perceived performance, we have conducted correlation analyses. The objective change was calculated by subtracting the pre-intervention value from the post-intervention value in every condition; thus, positive values refer to a performance improvement. Perceived and real change (i.e., difference scores) did not correlate in standard (*rho* = −0.10, *p* = 0.931), proprioceptive (*rho* = 0.11, *p* = 0.921), and visual (*rho* = 0.114, *p* = 0.319) conditions; a weak significant negative correlation was found for the vestibular condition (*rho* = −0.268, *p* = 0.018).

## Discussion

In this experimental study, we have investigated placebo and nocebo effects on postural stability. For this purpose, participants were administered an inert sports cream containing no active ingredients, with the information that it would increase (placebo group), decrease (nocebo group), or not affect (control group) their postural stability. Although the placebo group expected a better performance than the nocebo group, no differences between groups in any measure of postural stability were found. Participants’ expectations were positively associated with perceived but not actual postural stability. Finally, perceived performance was independent of actual performance for all postural stability tests.

Results were different for actual (i.e., assessed with the BTrackS™ Balance Plate) and perceived (subjective) performance. No difference was found between the groups in any of the postural stability tests for the actual performance. Consequently, no placebo or nocebo effects were found. Participants performed best in the standard condition of the trial, followed by the proprioceptive and visual conditions, while the worst performance occurred in the vestibular condition. This pattern is in accord with our knowledge concerning the difficulty levels of the four tests (Goble et al., 2020). In the standard condition, information from all three sensory modalities (visual, proprioceptive, vestibular) is available, which improves participants’ performance. Reliance on proprioception or vision still leads to relatively good performance, as these are the typically used sensory modalities under normal circumstances. Finally, contribution of information from the vestibular system is generally considered inferior to the other modalities (Goble et al., 2020). Also, participants generally performed better in the post-intervention measurement, which may reflect the effect of practice. Overall, these findings suggest that our participants tried to do their best during the tests.

A possible explanation for the lack of placebo/nocebo effects is that manipulation of expectancies was not as powerful as in other studies (Russell et al., 2022; Villa-Sánchez et al., 2019). Although there was a significant difference in expectancies between the placebo and the nocebo group, neither of them differed from the control group. As interaction between the experimenter and the participant plays a role in the development of placebo effects (Beedie et al., 2018), limited interaction (i.e., instructions were administered via a recording) might have decreased expectations. Also, the agent we applied (a cream) might have been a less powerful placebo than the agents (electrical treatment of a muscle, ingestion of a pill) used in previous studies (Russell et al., 2022; Villa-Sánchez et al., 2019). This explanation is partly supported by the findings of a study (Szabo et al., 2013), indicating higher perceived effectiveness of internally applied placebos (pills, drinks) than external treatments (lotion, gel) in the improvement of physical abilities. Concerning transcutaneous nerve stimulation used in the study of Villa-Sánchez et al. (2019), it evoked a slight sensation of the skin, which might have improved its credibility. Also, the index used to characterize stability in the current study (i.e., Total COP Path Length index), does not differentiate between the medio-lateral and anterior–posterior components of (in)stability; in the previous two studies, typically the latter component was impacted by the respective intervention. Considering the fact that the size of the sample was considerably larger (overall 78 participants in three groups) in our study than in previous studies (*n* = 30 in two groups; *n* = 42 in three groups), whereas the assessed populations (young individuals) were similar, the background of this null finding is certainly worthy of further investigation. Finally, it is important to emphasize that expected performance did not predict actual performance in our study.

With respect to perceived performance, the placebo group showed higher values than the other two groups; however, these differences disappeared when the covariates were included in the analysis. This pattern suggests that certain covariates (most likely expectation with a strong significant effect) substantially impacted participants’ internal assessment of performance.

The post hoc correlation analysis confirms the existence of an almost complete dissociation between actual and perceived performance in the balancing tests. For the vestibular condition, a weak negative association was found, such as the more the performance deteriorated in the vestibular condition, the greater performance improvement participants perceived. The dissociation between actual and perceived performance is not a novel finding. For example, when investigating the role of inhaled essential oils (e.g., peppermint, rosemary, eucalyptus), Köteles et al. (2018) found that only perceived but not objective spirometry performance was influenced by the expectations. Similar findings were obtained concerning the perception of heart rate, sustained attention, and alertness (Babulka et al., 2017). For cognitive performance, a similar dissociation was revealed. For example, Schwarz and Buchel (2015) found that placebo and nocebo treatments only affected perceived performance but not actual performance on the Flanker task (assessing cognitive response inhibition). Also, in the study of Winkler and Hermann (2019), only perceived but not actual cognitive performance was influenced by placebo and nocebo treatments. Our results align with these studies; if one’s knowledge, previous experience, or perceptual abilities are insufficient to estimate the actual condition accurately, expectation substantially shapes (biases) perception, which leads to a dissociation between the actual and perceived state (Köteles, 2021). These findings, again, have a potential implication for clinical rehabilitation settings because the perception of better performance could fuel motivation in adherence to various treatments. Indeed, based on competence motivation theory (Harter, 1978), people tend to pursue activities if they believe they can perform those behaviors successfully.

Another goal of this research was to investigate the possible effect of psychological state and trait characteristics in placebo and nocebo effects, such as state and trait anxiety, optimism, holistic thinking, persistence, and motivation to cooperate. In this study, we have found that these characteristics do not really influence the magnitude of the placebo and nocebo effect with respect to postural stability. Although two significant effects were obtained (persistence in the proprioceptive test and optimism in the vestibular test), the background of the interactions between psychological traits and balancing test types is difficult to explain. Still, research on interactions between psychological characteristics and more effective placebo treatments appears a potentially important research direction.

It should be noted that several factors can influence the outcome of a placebo and nocebo intervention in motor performance. For example, the type of agent, characteristics of the investigated population, the research design, and the exact aspect of motor performance can all impact the results (Horváth et al., 2021; Hurst et al., 2019). How the perceptual properties of various agents interact with personality and cognition is the subject of basic research, which are fueled by the results of applied research as the current one. Nomothetic research is based on “majority” or general findings, but basic research is the path to understanding why some people respond to a placebo/nocebo intervention while others do not. Furthermore, it is the task of basic research to explore why some perceived as weak agents cannot elicit placebo/nocebo responses, while internally applied agents can.

Here, we used a sham sports cream administered with verbal instructions. It is possible that other agents (i.e., a pill, a green drink, or sham transcutaneous stimulations) or instructions worded differently (de la Vega et al., 2017) could have evoked different findings. Indeed, a study has shown that while a fictive green drink was perceived as the best strength, endurance, and concentration-improving agent, externally applied agents like a fictive white lotion or green gel were perceived as the least efficient (Szabo et al., 2013). Still, we opted for an externally applicable agent to avoid any potential harm associated with ingestible substances. Future studies should replicate this work with agents with more credibility, like placebo pills, sham electric or magnetic stimulation, or sham (saline) injection to see if the results would be similar. This recommendation is based on a study showing that arthroscopic surgery results in patients with a degenerative medial meniscus tear were not superior to a sham (placebo) surgical procedure (Sihvonen et al., 2013). Therefore, more powerful placebos might have different effects than a sham cream used in this study.

### Limitations

This laboratory work has limitations too. First, we investigated a sample of young individuals (healthy university students); thus, our findings may not be generalized to older people (Goble et al., 2020) and those needing movement or postural rehabilitation. Second, because we applied a passive agent (without any active ingredient), it is still possible that placebo and nocebo instructions administered with an active placebo might have been more effective (de la Vega et al., 2017; Szabo et al., 2018). Third, we used an inert white cream, which in a previous study (Szabo et al., 2013) was perceived as less effective than ingestible fictive agents. Therefore, future studies should replicate this work with other agents. Furthermore, in this study, the control group received the agent with ineffective (neutral) verbal instruction. This intervention may be another limitation because it is possible that such an instruction still generated different expectations in different participants.

## Conclusions

Postural stability appears not susceptible to placebo and nocebo influences if the placebo agent is an inert white cream and the interaction between researcher and participants is limited. However, a dissociation between actual and perceived performance was observed, where expectations impacted *subjective* but not objective performance. Based on (perceived) competence motivation, these findings might have implications for motivation for interventions aimed at improving postural stability.

## Supplementary Information

**Additional file 1:** Supplementary material.

## Data Availability

https://osf.io/6fbxs/
