# Supplementary material for: Are there placebo or nocebo effects in balancing performance?
Source: Cogn Res Princ Implic. 2023 Apr 24;8:25. doi: 10.1186/s41235-023-00476-z (PMC10126169; doi:10.1186/s41235-023-00476-z)
Supplement: Supplementary file 1 — Additional file 1: Supplementary material. [file 41235_2023_476_MOESM1_ESM.docx]

**Supplementary materials 1**

**Placebo group instruction**

The cream is free from any odorant and colorant substance, so we can investigate the pure effect of the active substance. According to the regulations, we provide information about the expected effects of the cream. The agent has a warming-up effect and is typically used to enhance sports performance. It increases the blood flow in the muscles and improves the conduction of impulses between neurons and muscle cells. Altogether, these effects can improve muscle performance, that have a positive effect on movement regulation, including balancing ability. Do not use the cream if you are diagnosed with high blood pressure (hypertension). Please cream both of your feet (muscles of the thigh, knee-joint, calf, shin, and ankle-joint), each with one dose. Do not use the cream on and around wounds on your skin. Please massage the cream for about 1 minute per foot. The absorption time is about 5 minutes. After that, we are going to measure your balancing ability again. The cream does not contain any doping or prohibited ingredients.

**Nocebo group instruction**

The cream is free from any odorant and colorant substance, so we can investigate the pure effect of the active substance. According to the regulations, we provide information about the expected effects of the cream. The agent has a slight anesthetic and anti-inflammatory effect and is typically applied after muscle injuries (muscle strain, bruise, pulled muscle). Its use is not recommended in the absence of injury as it negatively impacts motor performance. It decreases the blood flow in the muscles and slows down the conduction of impulses between neurons and muscle cells. Altogether, these effects can decrease muscle performance, that have a negative effect on movement regulation, including balancing ability. Do not use the cream if you have a diagnosed blood clotting disorder. Please cream both of your feet (muscles of the thigh, knee-joint, calf, shin and ankle-joint), each with one dose. Do not use the cream on and around wounds on the skin. Please massage the cream for about 1 minute per foot. The absorption time is about 5 minutes. After that, we are going to measure your balancing ability again. The cream does not contain any doping or prohibited ingredients.

**Control group instructions**

You were assigned to the control group, so the cream does not contain any active substance. The cream is free from any odorant and colorant substance. According to the regulations, we provide information about the expected effects of the cream. The cream does not affect the blood flow in the muscles and does not affect the speed of the conduction of impulses between neurons and muscle cells. Altogether, it does not affect muscle performance and movement regulation, including balancing ability. The cream can be used by everybody. Please cream both of your feet (muscles of the thigh, knee-joint, calf, shin, and ankle-joint), each with one dose. Do not use the cream on and around wounds on the skin. Please massage the cream for about 1 minute per foot. The absorption time is about 5 minutes. After that, we are going to measure your balancing ability again. The cream does not contain any doping or prohibited ingredients.
